# Advancing the Science of Tick and Tick-Borne Disease Surveillance in the United States

**DOI:** 10.3390/insects10100361

**Published:** 2019-10-19

**Authors:** Samantha M. Wisely, Gregory E. Glass

**Affiliations:** 1 Department of Wildlife Ecology and Conservation, 110 Newins Ziegler Hall, University of Florida, Gainesville, FL 32611, USA; 2 Emerging Pathogens Institute, University of Florida, Gainesville, FL 32611, USA; gglass@epi.ufl.edu; 3 Department of Geography, University of Florida, Gainesville, FL 32611, USA

**Keywords:** citizen science, National Ecological Observatory Network, One Health, species distribution modeling, state-space modeling, surveillance

## Abstract

Globally, vector-borne diseases are an increasing public health burden; in the United States, tick-borne diseases have tripled in the last three years. The United States Centers for Disease Control and Prevention (CDC) recognizes the need for resilience to the increasing vector-borne disease burden and has called for increased partnerships and sustained networks to identify and respond to the most pressing challenges that face vector-borne disease management, including increased surveillance. To increase applied research, develop communities of practice, and enhance workforce development, the CDC has created five regional Centers of Excellence in Vector-borne Disease. These Centers are a partnership of public health agencies, vector control groups, academic institutions, and industries. This special issue on tick and tick-borne disease surveillance is a collection of research articles on multiple aspects of surveillance from authors that are affiliated with or funded by the CDC Centers of Excellence. This body of work illustrates a community-based system of research by which participants share common problems and use integrated methodologies to produce outputs and effect outcomes that benefit human, animal and environmental health.

## 1. Introduction

Globally, vector-borne diseases are an increasing public health burden. In the United States, >75% of all vector-borne diseases are tick-borne, and the number of reported cases of tick-borne infections doubled between 2004 and 2016. More than 40,000 cases of tick-borne disease have been reported since 2011, yet there is likely an eight-to-ten fold higher number of cases than are reported [1]. Despite underreporting, increases in tick-borne diseases have been documented for Lyme disease [2], human babesiosis [3], rickettsiosis [4], and ehrlichiosis [5].

In addition to the spread and increase of the most common tick-borne diseases, new disease-causing pathogens such as *Borrelia miyamotoi*, a relapsing fever group of *Borrelia* [6] and *Ehrlichia muris eauclairensis* (Xu et al., 2018) have been discovered. Viral agents such as Powassan virus have shown a rapid increase in human cases [1], and previously undescribed viruses such as Bourbon virus and Heartland virus have been found in rapidly expanding populations of *Amblyomma americanum* [7]. It is evident that the pathogen landscape in the U.S. and elsewhere is rapidly changing, and the need to address this change is acute.

The causes of this global increase in tick-borne diseases are multi-factorial but have been attributed to the characteristics defined by the current Anthropocene geologic epoch. Climate change, land cover change, land use change, population growth, global transportation, global trade, and socio-economic forces have converged to alter the biogeophysical composition of our planet, and these alterations have catalyzed the increase in vector-borne diseases [8,9]. Despite these broad patterns of converging factors implicating global change in the rise of vector-borne diseases, the mechanisms underlying transmission and ultimately prevention remain, in many cases, elusive due to the complex nature of vector-borne disease epidemiology. It is within these murky details that the value and necessity of vector and vector-borne disease surveillance become evident.

The United States Centers for Disease Control and Prevention (CDC) has recognized this national need for resilience to the vector-borne disease burden and has called for increased partnerships and sustained networks to identify and respond to the most pressing challenges that face vector-borne disease management. As part of that response, the CDC created a network of five nationwide Centers of Excellence that provides a focus on workforce development, communities of practice that increase local and state capacities to manage the disease burden and its causes, and applied research into prevention and control [10]. To achieve these goals, Centers are a partnership of public health agencies, vector control groups, academic institutions, and industries. This special issue on tick and tick-borne disease surveillance is a collection of research articles on multiple aspects of surveillance from authors that are affiliated with or funded by the CDC Centers of Excellence. While not representing all aspects of vector-borne disease research funded by this CDC partnership, surveillance is recognized as a key aspect of a functioning public health response.

## 2. The Science of Surveillance and Its Application

Surveillance is one of the pillars of infectious disease management. In its most basic form, surveillance, whether passive or active, can provide early warnings of newly emerging pathogens [11] such as the discovery of *Borrelia miyamotoi* found in a surveyed population of *Ixodes scapularis* in 2001. Twelve years later, the first human case attributed to this pathogen occurred [12] and was likely detected by health professionals because of the earlier surveillance efforts. Likewise, the incursion of exotic vectors such as the Asian longhorned tick, *Haemophysalis longicornis*, was first identified in New Jersey in 2017, which created awareness of this potentially devastating human and animal disease vector [13]. Increased vigilance and enhanced surveillance as a result of this finding has suggested that the tick is established in multiple eastern states [14].

Surveillance as a field of science receives relatively little consideration. Two papers in this special issue report on the practical evaluation of surveillance methodology. Glass et al. [15] used a literature review of tick surveys in Florida to assess the myriad of different surveying techniques, and compared surveillance outcomes to illustrate the importance of choosing the best surveillance technique a priori in order to meet the objectives of surveillance. They then provided a methodology and rationale for the type of sampling required to generate species distribution models. The resulting discussion makes recommendations for the establishment of surveillance that can lead to a better understanding of the biogeography of medically important ticks in ecologically diverse regions like Florida.

The second paper that evaluates the science of surveillance [16] demonstrates how data acquired from passive surveillance influence subsequent aspects of targeted intervention, treatment, and public health. Oftentimes, surveillance analyses, such as species distribution models (SDMs), input data from biased surveys because they are the best available data. Though SDM researchers warn against the impacts of these flaws, few studies have compared how much of an impact these biases have. Kessler and colleagues [16] compared data from a ‘typical’ citizen science survey with a standardized data collection for the same tick species in Florida. The results illustrated a discrepancy in the predicted distribution associated with biased data. Nonetheless, biased data sets still provide important information on where vectors do occur, but they are limited in their utility for extrapolating results to other places.

An example of keen a priori surveillance planning to achieve an objective can be found in the study by Egizi et al. [17]. While acknowledging that active surveillance requires funding and infrastructure that most agencies lack, these authors harnessed the infrastructure that New Jersey has in place for vector control, the New Jersey mosquito control community, and demonstrated that with minimal resource investment, they could perform a standardized tick survey for an understudied and underappreciated disease vector, *Dermacentor variablis*. The result was an increased knowledge of the distribution of this tick and other species that inhabit grasslands and meadows, as well as a working protocol for engaging vector control communities in active surveillance of ticks.

While the majority of the public health burden due to tick-borne diseases is in the northeastern US and increasingly in the upper Midwest US, other regions struggle with less well understood tick-borne diseases. Diseases such as southern tick-associated rash illness (STARI) have unknown pathological agents, and many diseases such as ehrlichiosis and rickettsiosis have multiple aetiological agents that are only beginning to be understood [18]. Two papers in this special issue provide survey results for tick-borne pathogens in previously under-surveyed areas of the United States: Mendell et al. [19] conducted a targeted survey in a public space in Texas, while De Jesus et al. [20] conducted a statewide survey of bacterial tick-borne pathogens throughout Florida. These studies took a broad approach to molecular screening in questing ticks, using assays that detected multiple related bacterial species. Surveillance of this type can help piece together the epidemiological and aetiological puzzle for poorly understood tick-borne diseases. As with other forms of surveillance, these studies also help to inform the public as to risks associated with outdoor activities and provide baseline data for infectious disease clinicians who struggle to maintain vigilance of emerging tick-borne disease threats like Powassan virus [21] and Lyme disease [22].

While the aforementioned surveys all used active surveillance to drag, flag, or attract ticks with CO_2_, passive surveillance can provide additional insight not available using the above-mentioned surveillance methods. Lee et al. [23] formed a network of collaborators that included veterinary offices, animal shelters and wildlife rehabilitation centers throughout Wisconsin to passively survey for ticks attached to companion animals and wildlife. The resulting survey increased the known distribution of certain tick species in Wisconsin and established a baseline for future surveillance. In addition, the network of colleagues established in this study has the potential to build social capital that can be leveraged to sustain surveillance efforts and increase public health awareness.

## 3. Leveraging Surveillance Data to Further Understand Tick Biology, Distribution, and Management

In addition to identifying pathogen occurrence or defining vector communities, surveillance provides insight into the environmental factors that drive vector population processes. Climate change has been predicted to alter vector-borne disease dynamics [24], and for some pathogens like *Borrelia burdorferi*, climate warming is anticipated to facilitate sylvatic transmission [25] due to increased overwinter survival [26,27]. Using these insights, Linkse et al. [28] took a mechanistic approach to understanding the both broadscale and fine-scale environmental processes that drive overwinter survival in the vector of *B. burgdorferi* and *Ixodes scapularis*. Using replicate mesocosm experiments, they showed that leaf litter, more than snow accumulation, facilitates the overwinter survival of larvae, which has practical land management applications for both public and private landholders.

Ultimately, it is surveillance data that are necessary for modeling the dynamics and distributions of ticks and tick-borne diseases [29], but standardized surveillance data collected over multiple years are difficult to sustain due to a lack of consistent funding or political will. Exceptions, however, exist. The National Science Foundation National Ecological Observatory Network (NEON) is a nationwide survey and monitoring effort that is standardized across 81 sites in the United States. The mission of NEON is to provide long-term biogeophysical data in the continental US to better understand how global changes to the environment affect ecological processes. As part of its organismal sampling efforts, NEON collects ticks in a standardized fashion. Klarenberg and Wisely [30] utilized these data from one site to demonstrate the utility and power of this type of surveillance to model tick population dynamics using a state-space modeling approach. They showed that even a five year dataset demonstrates changes in abundance over time and illustrates the potential power of this nationwide government monitoring effort.

In addition to population dynamic modeling, robust species distribution models can be created from surveillance datasets. Kessler et al. [31] utilized three years of systematically collected data to generate ensemble SDMs that included both presence and “true” absence data to predict the distribution of medically important tick species throughout Florida. Importantly, the a priori spatial sampling considerations described in Glass et al. [15] were utilized in this research to address the design shortcomings of many modeling efforts. The results of the modeling exercise illustrate where human risks of encountering ticks is high, and the results can therefore be used to target human interventions.

Species distribution models can also be used to indicate where targeted surveillance should occur for potentially invasive tick species. Pascoe et al. [32] used geographic records of four *Amblyomma* tick species found in the Americas to model their potential distribution and invasion potential into California. They demonstrated that while some species may have the ability to persist in California, the climate is not conducive for other species. The resulting maps indicated areas where invasion potential is high and therefore should be targeted for enhanced surveillance.

## 4. Conclusions

As human cases of tick-borne disease continue to rise in the United States, the CDC urges local monitoring and surveillance in order to manage vector species and educate health care workers and the public about local disease risks. As evidenced by the collection of publications in this special issue on “Tick and Tick-borne Disease Surveillance,” effective surveillance and useful products resulting from surveillance efforts require collaboration among stakeholders with expertise in diverse disciplines. By incorporating humans, animals, and the environment, surveillance inherently becomes a One Health enterprise [33] that places the science and management of vector-borne diseases in a broader socio-ecological context. This holistic approach provides for a community-based system of research by which participants share common problems and use integrated methodologies to produce outputs and effect outcomes that benefit human, animal, and environmental health.
